# Editorial: Performance optimization in racket sports: The influence of psychological techniques, factors, and strategies

**DOI:** 10.3389/fpsyg.2023.1140681

**Published:** 2023-02-02

**Authors:** Nicolas Robin, Toru Ishihara, Emma Guillet-Descas, Miguel Crespo

**Affiliations:** ^1^Laboratory ACTES (EA 3596), Sport Sciences Faculty, University of Antilles, Pointe-à-Pitre, France; ^2^Graduate School of Human Development and Environment, Kobe University, Kobe, Japan; ^3^Laboratory of Vulnerabilities and Innovation in Sport, University of Claude Bernard Lyon 1, Univ Lyon, Lyon, France; ^4^International Tennis Federation, London, United Kingdom

**Keywords:** performance, training, competitions, tennis, table tennis, padel, badminton

Research and empirical work have revealed the importance of psychological or mental skills factors and strategies in improving athletic performance, especially in racket sports (Cece et al., 2020). The aim of this Research Topic was to bring together articles in which psychological techniques, factors, or strategies are discussed, used or tested in order to improve the performance in racket sport such as tennis, table tennis, badminton and padel.

Performing sport competitions usually generates psychological effects that can affect the psycho-emotional resources of athletes (Di Corrado et al., 2021). For example, Rodríguez-Cayetano et al. showed how precompetitive anxiety is one of the most important psychological factors in relation to padel performance and how it can vary according to competitive level and gender. According to Weinberg and Gould (1996), anxiety could be defined as a negative psycho-emotional mental state characterized by the manifestation of nervousness and worry, in which there is both somatic and cognitive components. Studies showed that anxiety can affect information processing and attentional control (e.g., Cocks et al., 2016), but more ecological validation in sport context, particularly in racket sports, is needed. That's why Ren et al. realized a study in which expert and non-expert table tennis players had to anticipate the serves of opponents under dynamic task constraints using a temporal occlusion paradigm. There findings showed that anxiety negatively influence attention and performance in athletes.

Other emotional response, such as the stress caused by physical or social daily stressors and psychological constraints (Martinent et al., 2020) can negatively influence performance in competitive sports (Lazarus, 2000) and may lead to athlete burnout (Martinent et al., 2014). Martinent et al. investigated the role of stress, recovery and coping on elite table-tennis players' burnout symptoms in considering the roles of contextual and individual factors. Athlete burnout can be defined as a chronic psychological syndrome, manifesting as an emotional and a physical exhaustion, a reduced sense of accomplishment and a devaluation of sport (Raedeke and Smith, 2001). Li et al. (2018) evoked that burnout may be a risk factor of sleep problems among athletes. However, sleep duration and quality can also influence athletic performance (e.g., Kirschen et al., 2020). Indeed, Han et al. evaluated the relationships of sleep inconsistency and duration with tennis competitive performance in soft tennis players and recommended to maintain regular sleep in daily life.

The analyzes made from the observation of competitions, generally with international level players, are also very useful to better understand factors that can influence performance in racket sports. For example, Wang explored the relationship between the scoring structure and the win or loss of a match to better understand what keeps international badminton players competitive. In addition, Sheng et al. investigated the effects of the context-related (e.g., importance of set or rally, game status or result against serve) and technical (e.g., defensive action, forehand or overhead strokes) variables of last strokes in rallies in elite matches. Finally, Kuroda et al. realized a study to better understand the association among time-course changes in the ratings for perceived exertion, the executive functions and the percentage of points won when playing tennis by including the second serve accuracy.

The results of these researches make it possible to improve training intervention or match tactics and implement performance optimization strategies. For example, integrating into research showing beneficial effects of using motor imagery strategy in tennis to improve performance (see Robin and Dominique, 2022 for a review), Robin et al. investigated the combination of this mental strategy with self-talk, in the first serve of skilled tennis players. The authors particularly recommend that tennis players, before performing a particular type of service, imagine seeing the trajectory of the ball and the area they would like to reach in the service box. Koya et al. also suggested the use of physical measurements to improve service performance. In addition to physical strength training, the authors suggested paying particular attention to the depth of the impact point to improve the speed of first serve balls.

The studies previously evoked and which you will find in this Research Topics entitled: “*Performance optimization in racket sports: The influence of psychological techniques, factors, and strategies*,” in addition to providing new knowledge in the fields of table tennis, padel, badminton and tennis, offer applied recommendations and suggestions to coaches and players as well as new research perspectives.

## Author contributions

All authors listed have made a substantial, direct, and intellectual contribution to the work and approved it for publication.
